# Absolute income is a better predictor of coverage by skilled birth attendance than relative wealth quintiles in a multicountry analysis: comparison of 100 low- and middle-income countries

**DOI:** 10.1186/s12884-018-1734-0

**Published:** 2018-04-16

**Authors:** Gary Joseph, Inácio C. M. da Silva, Günther Fink, Aluisio J. D. Barros, Cesar G. Victora

**Affiliations:** 1 International Center for Equity in Health; Post-Graduate Program in Epidemiology, Federal University of Pelotas, Rua Marechal Deodoro, 1160, 3o andar, Pelotas, RS 96020-220 Brazil; 2 Department of Global Health and Population, Harvard School of Public Health, 665 Huntington Avenue, Building 1, Room 110, Boston, MA 02115 USA

**Keywords:** Birth attendance, Institutional delivery, Household income, Low and middle-income countries

## Abstract

**Background:**

Having high-quality data available by 2020, disaggregated by income, is one of the Sustainable Development Goals (SGD). We explored how well coverage with skilled birth attendance (SBA) is predicted by asset-based wealth quintiles and by absolute income.

**Methods:**

We used data from 293 national surveys conducted in 100 low and middle-income countries (LMICs) from 1991 to 2014. Data on household income were computed using national income levels and income inequality data available from the World Bank and the Standardized World Income Inequality Database. Multivariate regression was used to explore the predictive capacity of absolute income compared to the traditional measure of quintiles of wealth index.

**Results:**

The mean SBA coverage was 68.9% (SD: 24.2), compared to 64.7% (SD: 26.6) for institutional delivery coverage. Median daily family income in the same period was US$ 6.4 (IQR: 3.5–14.0). In cross-country analyses, log absolute income predicts 51.5% of the variability in SBA coverage compared to 22.0% predicted by the wealth index. For within-country analysis, use of absolute income improved the understanding of the gap in SBA coverage among the richest and poorest families. Information on income allowed identification of countries – such as Burkina Faso, Cambodia, Egypt, Nepal and Rwanda – which were well above what would be expected solely from changes in income.

**Conclusion:**

Absolute income is a better predictor of SBA and institutional delivery coverage than the relative measure of quintiles of wealth index and may help identify countries where increased coverage is likely due to interventions other than increased income.

**Electronic supplementary material:**

The online version of this article (10.1186/s12884-018-1734-0) contains supplementary material, which is available to authorized users.

## Background

The Sustainable Development Goals (SDGs 2030) were adopted by the United Nations in 2015. Target 17.18 requires the enhancement of country capacity to produce high-quality, timely and reliable data disaggregated by income and other stratifiers, by 2020 [1]. In spite of the SDG recommendation, few low and middle-income countries (LMICs) collect systematic data on household income [2]. Non-monetary income, such as in-kind gifts or trading, is generally hard to measure, and informal employment often results in transitory, or irregular incomes that are hard to quantify. As a result, income may fluctuate more over time than other socioeconomic indicators such as the wealth index that is based on assets, dwelling materials and access to electricity and sanitation [3]. In addition, reporting income is a sensitive topic, and interviewees are often reluctant to divulge such information [2]. Although the association between income and health indicators is strong in most societies [4], it is not always possible to establish whether low income led to poor health, or whether poor health reduced income, for example due to unemployment or absenteeism [5, 6].

Given the difficulties presented above, direct estimates of incomes and poverty are generally not available in health surveys conducted in LMICs, and most studies have focused their attention on the relative socioeconomic position (SEP) of the household using the wealth index [3]. Wealth indices are usually calculated through principal component analysis of variables related to household infrastructure and asset ownership such as television, cars, bicycle, access to clean water, landholdings, among others [2, 3, 7]. Different from income, the variables needed to produce these indices are easy and reliable to collect, and the index is simple to compute. They have helped to increase the prominence of health inequalities on the global health agenda [8–11]. Although there have been attempts to calculate universal wealth indices for cross-country comparisons [12], most published analyses rely on asset indices for assessing relative SEP within a survey, in contrast to income which has an absolute value and can therefore be compared across as well as within surveys [3].

Harttgen and Vollmer showed that household income can be approximated well through a combination of aggregate data on income at country level and income inequality as well as micro-data on relative wealth [13]. Recent work by Fink et al. suggests that predicted income created through this approach is markedly superior to relative wealth quintiles when predicting stunting levels across countries and over time [14].

In the present paper, we use this newly available approach (data on household income is available for download at: http://databank.worldbank.org/data to assess the extent to which income differentials can predict skilled birth attendance (SBA) and institutional delivery coverage in LMICs, one of the least equitable indicators of intervention coverage (SBA) in maternal and newborn health [10, 15]. We then provide examples of how information on absolute income can contribute to interpreting time trends in SBA coverage by wealth in selected countries. Institutional delivery was used as a secondary outcome given it is also an important indicator of safe deliveries and has a more comparable definition across countries.

## Methods

Data for this analysis were retrieved from publicly available nationally representative health surveys carried out from 1991 to 2014, including the Demographic and Health Surveys (DHS), Multiple Indicator Cluster Surveys (MICS), and Reproductive Health Surveys (RHS). In this study, we used data from 293 surveys done in 100 LMICs for which information on SBA, wealth index and income data were available. All surveys used multi-stage cluster sampling designs to obtain nationally representative data. Standardized questionnaires were used to collect information from women of reproductive age living in the sampled households. DHS, MICS and RHS collect information in LMICs only, and generally are implemented by national census or statistics agencies. Ethical approval was the responsibility of the institutions in charge of each survey. More details on DHS, MICS and RHS are available elsewhere [16–18].

Wealth was assessed by the wealth index divided into quintiles at the household level. The wealth index is pre-calculated in all DHS and MICS surveys and most RHS surveys. It is estimated through principal components analysis, using variables on household assets, building materials of the dwelling and access to utilities such as electricity, water and sanitation. Quintile 1 (Q1) represents approximately the poorest 20% of households in the surveys sample and quintile 5 (Q5), the 20% richest.

Data on household income were retrieved from Fink et al. [14]. As discussed in further detail in the original paper [14], household income distribution is computed based on national income levels and national income inequality data. National income per household level adjusted for consumption share, population size, and mean number of persons per household is obtained from the World Bank database of indicators. National income inequality comes from the Standardized World Income Inequality Database [19] – it gives a measure of how income is distributed across the country, and we assign dollar values (2011 purchase power parity adjusted international dollars) to each household wealth quintiles (from the original survey asset index) supposing the income distribution follows a lognormal distribution. More details about this process of calculating household income can be found elsewhere [14].

Given our focus on SBA, we restricted the analysis to female respondents with a live birth in the last 3 years in DHS and RHS. For MICS surveys, SBA information is only available for the last live birth in the previous 2 years. Women interviewed were aged 15 to 49 years in all DHS and MICS surveys, but some RHS surveys collected data on women aged from 15 to 44 years old (Albania 2002, Belize 1991, Moldova 1997, Costa Rica 1992 and Paraguay 1995).

Our outcome was SBA coverage, determined by asking the women “Who assisted with the delivery of <NAME OF THE CHILD>?”. Options included doctor, nurse, auxiliary nurse, midwife, traditional birth attendant (TBA), relative or friend, other, or no one. In our analysis, we defined SBA as a birth attended by a doctor, nurse, midwife or another cadre that the country recognized as skilled (auxiliary midwife, auxiliary nurse, community health officer), depending on their health programs and policies, and independently of the place of delivery.

We used cross-country and within-country analysis to explore the degree to which absolute income and relative wealth predict coverage using multivariate regression model and adjustment for clustering. We, also, used the actual mean wealth index scores for each quintile to indicate whether there are larger gaps between some quintiles than others, in a similar way to absolute income. The units of analysis were the five wealth quintiles in each survey, and all surveys were analyzed together (including countries with two or more surveys over time). SBA coverage and absolute income for each quintile were organized in single dataset. In the analyses of relative wealth, quintiles were coded as 1–5, and in the analyses of absolute income, quintiles were allocated the dollar value of their average income. In the cross-country analyses, country-specific intercepts were not fitted, in contrast to the within-country analyses in which an intercept (fixed effects) was included for each survey.

Given that SBA coverage is restricted to the 0–1 interval and many countries present high coverage, we used a logit transformation for SBA to avoid our linear model predicting values outside the valid range. The predicted values were back transformed graphic presentation.

To address the second objective of assessing how information on absolute income can contribute to an understanding of within-country inequalities, and of time trends in SBA coverage by quintile, we compared countries with contrasting levels of national income and varied patterns of inequality: Ethiopia (low-income country), Nigeria (lower middle-income country) and Namibia (upper middle income) [20]. We then assessed trends over time in three groups of countries according to trends [21] in the last two decades: countries that had improved their coverage by 40 or more percentage points; countries that had progress less than 10 percentage points, and countries that had no change in income. We performed the same analyses using institutional delivery (defined as a birth taking place in a hospital, health center, clinic, or doctor’s office) coverage in those countries, except for Costa Rica (1992) that had no data on delivery place. All analyses were carried out using Stata (StataCorp. 2013. Stata Statistical Software: Release 13. College Station, TX: StataCorp LP).

## Results

We analyzed a total of 293 surveys (196 DHS, 86 MICS and 11 RHS) conducted between 1991 and 2014 across 100 LMICS (See Additional file1: Table S1). Mean SBA coverage was 68.9% (SD: 24.2) ranging from 6.6% in Ethiopia (2000) to 100.0% in Belarus (2012). Mean institutional delivery coverage was 64.7 (SD: 26.6) and ranging from 4.1% in Bangladesh (1993) to 100.0% in Barbados (2012) and St Lucia (2012). Median daily household income in the same period was US$ 6.4 (IQR: 3.5–14.0) and ranging from US$ 1.2 in Ethiopia (2000) to US$ 35.5 in Barbados (2012).

Table 1 shows results from the linear regression analyses. Indicator variables for relative wealth predicts 22.0% of total variation in the coverage of SBA (Table 1, column 1) in the cross-country analysis, while the log-normalized income (model 3) explains 51.6% of the total variation. In contrast, the actual mean wealth scores predict only 12.8% of the variation in coverage (Model 3).

**Table 1:** Linear regression analyses to investigate how well relative quintiles, actual mean wealth index scores and absolute income (per quintile) predict SBA coverage (*N* = 1465 observations)

	SBA prevalence (coefficients expressed as percent point)						
Analysis level	Cross-country analysis			Within country analysis			
	Model 1	Model2	Model 3	Model 4	Model 5	Model 6	Model 7
Asset quintile 1	0 (reference) *p* < 0.001			0 (reference) *p* < 0.001			0 (reference) *p* = 0.139
Asset quintile 2	10.19 (1.05)			10.19 (1.17)			2.18 (3.93)
Asset quintile 3	18.66 (1.80)			18.66 (2.02)			5.36 (6.53)
Asset quintile 4	28.60 (2.23)			28.60 (2.49)			9.98 (9.23)
Asset quintile 5	40.04 (2.56)			40.04 (2.86)			11.79 (14.02)
Mean wealth scores		6.97 (1.82) *P* < 0.001			6.85 (3.29) *P* < 0.001		
Log income^a^			19.13 (1.24) *p* < 0,001			18.38 (1.31) *p* < 0.001	12.78 (6.13) *p* = 0.04
Survey specific intercepts	NO	NO	NO	YES	YES	YES	YES
R-squared	0.220	0.128	0.516	0.877	0.777	0,879	0,881

Robust standard errors in parentheses are clustered at the country level

^a^Income is expressed in 2011 purchasing power parity-adjusted international dollars. Model 1 and model 4: cross-country and within-country prediction of SBA coverage according to wealth quintiles. Model 2 and model 5: cross-country and within-country prediction of SBA coverage according to actual mean wealth scores. Model 3 and model 6: cross-country and within-country prediction of SBA coverage according to household income. Model 7: within-country prediction of SBA coverage according to wealth quintiles and household income

Models 4 to 7 include survey-specific intercepts (survey fixed effects) to produce within-country analyses. The total variation explained increases to approximately 88% with any combination of the asset and income variables (models 4–7). When we have both the relative and absolute measures in model 5, only the log-normalized income variable remain statistically significant (*p* = 0.04). In model 5, due to the correlation between wealth quintiles and the absolute income, the standard errors in are larger than those in models 3 and 4, but the increase is not so large to suggest that model 5 is unstable and cannot be interpreted. Similar results for all models were observed when the outcome was institutional delivery coverage (see Additional file 2: Table S2).

**Fig. 1:**
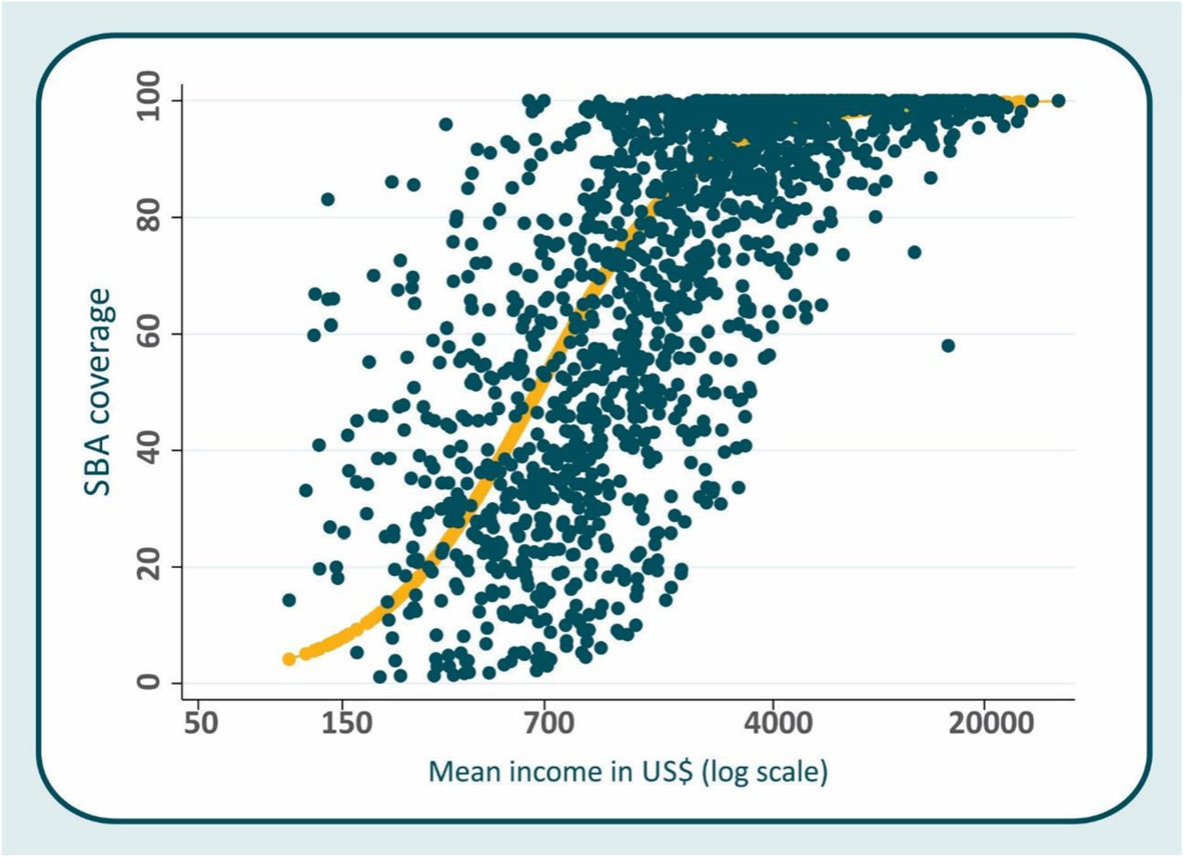
SBA coverage by log absolute income. Each dot is one quintile in each survey

Figure 1 shows the relationship between SBA coverage and log annual income, based on model 2, Table 1. Each dot represents one quintile in a given survey. The curve obtained though the linear model with logit transformation is S-shaped. When log annual income exceeds about US$7000, coverage becomes universal in most groups. The same relationship is also observed when we plotted the institutional delivery coverage against the log annual income (see Additional file 2: Figure S1).

**Fig. 2:**
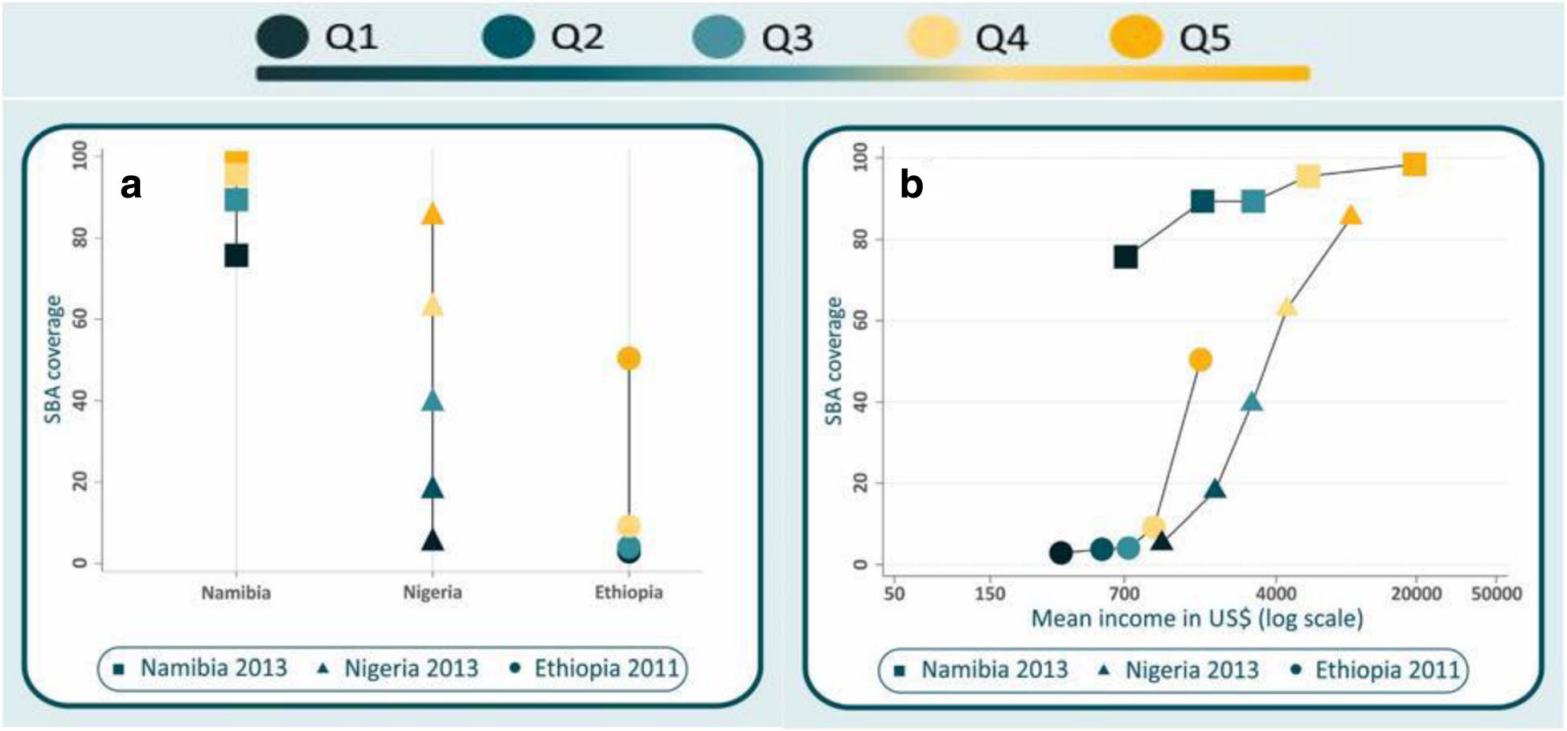
SBA coverage in Namibia, Ethiopia and Nigeria according to **a** wealth quintiles and **b** absolute income in the most recent survey

**Fig. 3:**
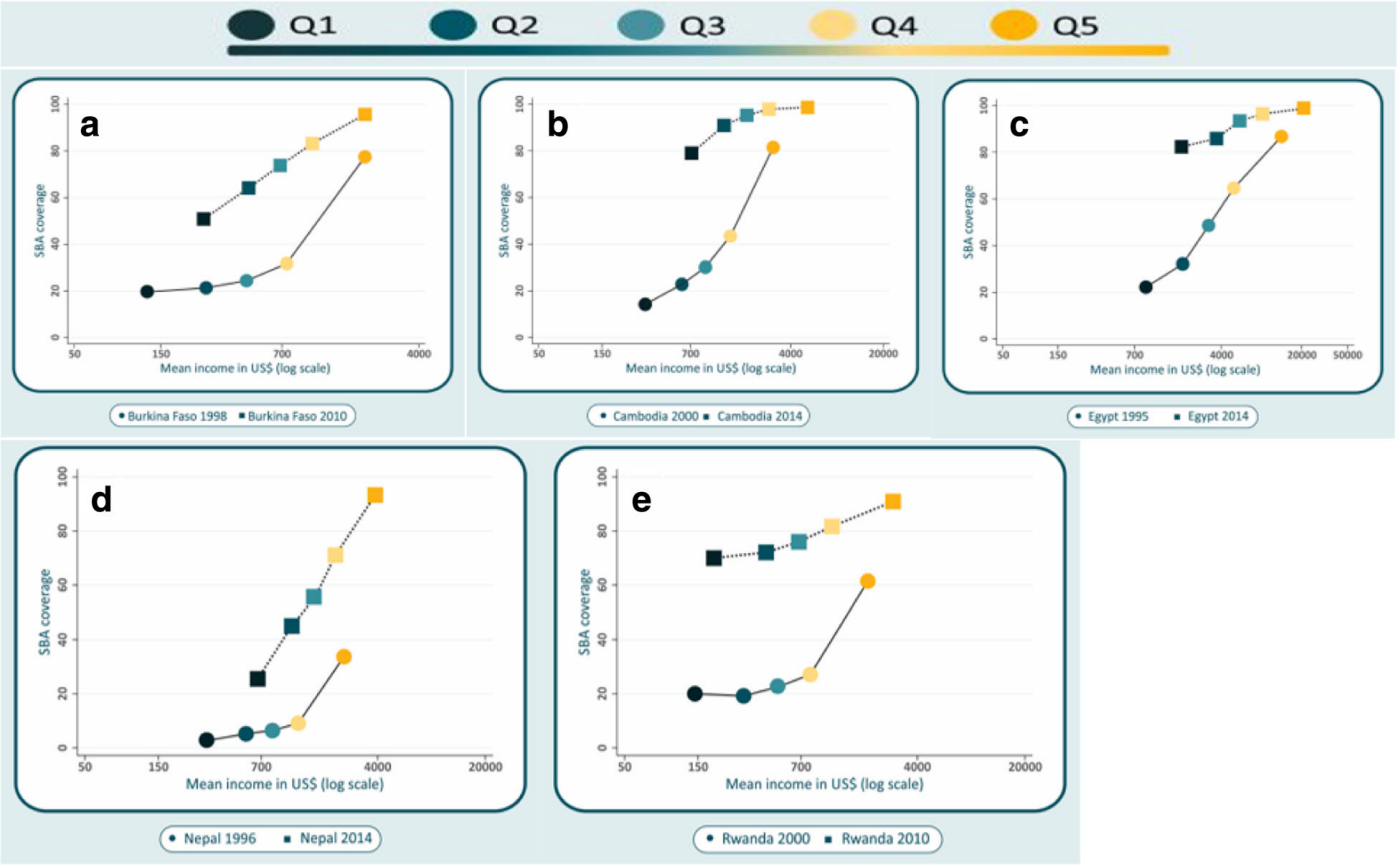
Five countries with increases in SBA coverage > = 40 percentage points over time: Burkina Faso, Cambodia, Egypt, Nepal and Rwanda

**Fig. 4:**
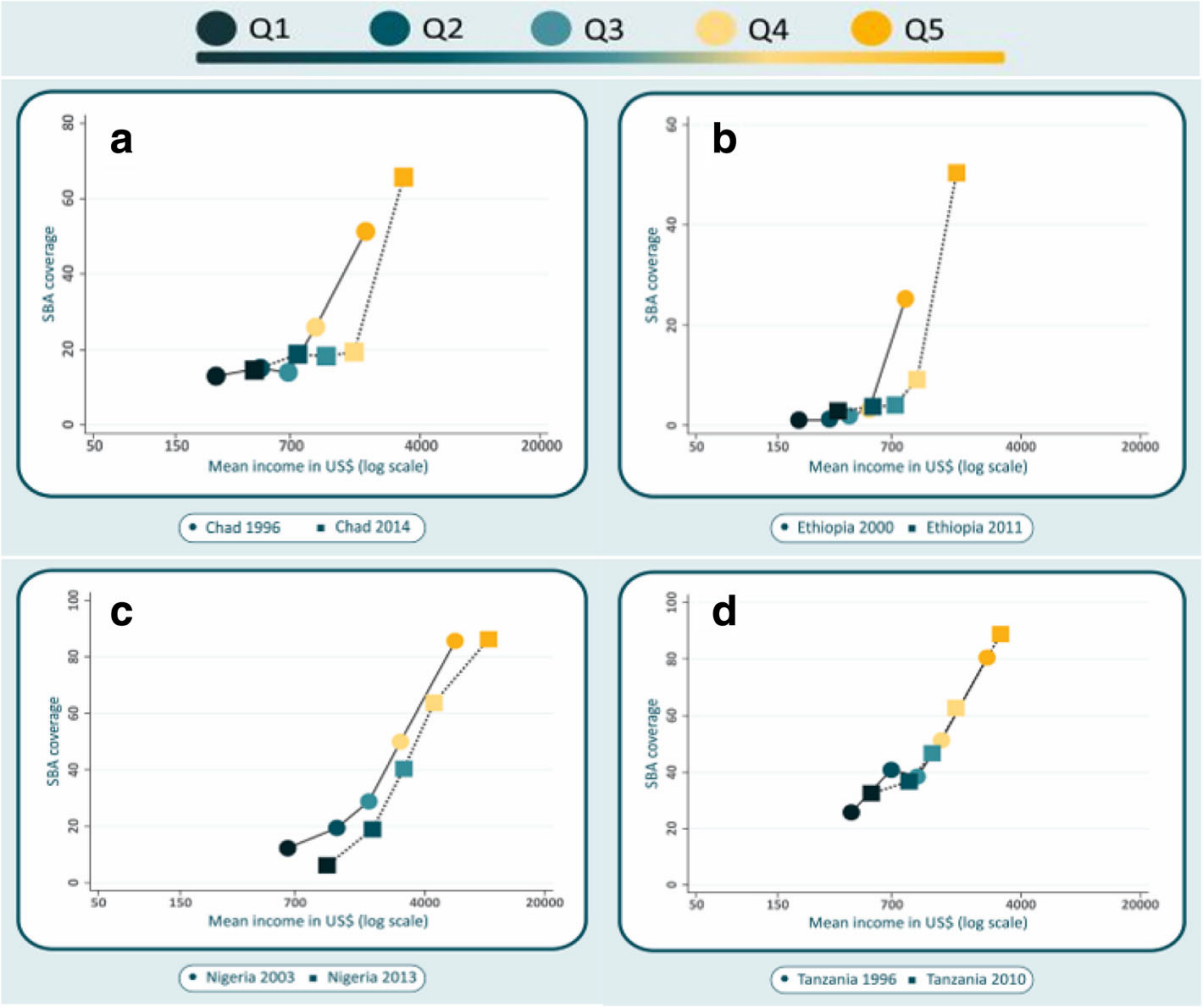
Four countries with increases in SBA coverage < 10 percentage points over 10 or more years: Chad, Ethiopia, Nigeria and Tanzania

**Fig. 5:**
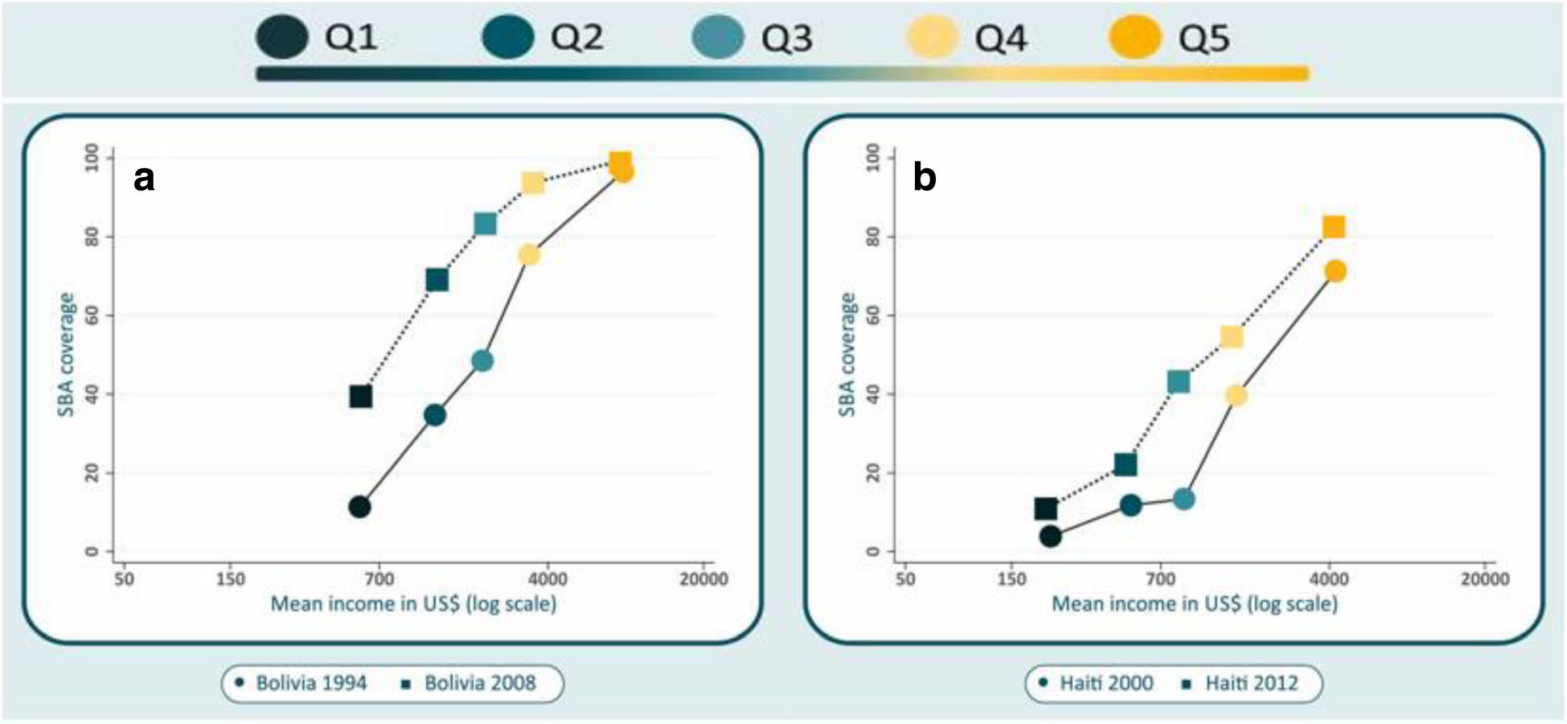
Two countries with no progress in household income over 10 or more years but with increase in SBA coverage (Bolivia, Haiti)

In order to illustrate the advantages of using absolute income, Figs. 2, 3, 4 and 5 compare this new approach with relative quintiles for selected countries. We also presented the same analysis using institutional delivery coverage in selected countries (see Additional file 2: Figures S2-S5). In Fig. 2, we show results from the most recent surveys of three countries in Sub-Saharan Africa with contrasting levels and patterns of inequality. Figure 2a is a traditional presentation of inequalities in coverage by relative quintiles (the “equiplot” www.equidade.org/equiplot), showing the widest gaps in Nigeria, followed by Ethiopia (where inequality is mostly due to the richest being well above the rest) and Namibia, where inequalities are largely due to the poorest quintile lagging behind. In contrast, Fig. 2b displays coverage corresponding to the absolute income in each quintile. In the three countries, incomes overlap only partially, with Ethiopian households in the top quintile having almost the same income than Nigerian households in the second quintile, and lower income than the Namibian in the second quintile. For any given income level, the coverage of SBA tends to be greater in Namibia compared to the Ethiopian or Nigerian households.

Figure 3 shows SBA coverage as a function of predicted income for the earliest and latest survey rounds in five countries with coverage increases of over 40%age points. In Cambodia, Egypt and Nepal, the average incomes of households in the poorest quintile in the last survey were higher than the average incomes in the second quintile in the earlier survey. In these countries, the relationship between household income and SBA coverage became less steep over time, as the top quintile reached universal coverage. Coverage in the poorest quintile was around 80% in the most recent surveys in these countries. In Burkina Faso and Nepal, incomes and SBA coverage increased for every quintile, but the slope has not changed, possibly because the richest quintiles have not yet reached universal coverage. In all five countries with national increases of over 40% points, coverage rose more rapidly than would be predicted from the change in income.

Figure 4 shows countries with small changes in national SBA coverage over time: Chad, Ethiopia, Nigeria and Tanzania. There were small increases in income over time for every quintile. Changes in SBA coverage seem to have been due to these slight increases in income. In Nigeria, income also increased but coverage declined for the poorest quintile – but not for the other four.

Figure 5 shows the results for countries where income hardly changed between two surveys at least 10 years apart. In Bolivia and Haiti, SBA coverage increased by 23.6 and 14.7 percentage points, respectively, in spite of lack of progress in income.

## Discussion

The approach proposed in this paper follows a series of previous attempts to allow tracking of absolute wealth changes over time using DHS data [22–24]. Rutstein et al. [22] as well as Staveteig and Mallick [24] have developed methods that allow to directly track changes in asset holdings over time. Hrushka and Gerkey [23] use a model very similar to the one presented here to estimate households absolute wealth holdings, but used data on income share, which is available for fewer countries than the Gini coefficient. The main advantages of the approach followed here is that it directly predicts consumption per capita for each household, which allows to directly assess the poverty status of each household. We opted to predict income levels for wealth quintiles, rather than attempt to predict them for individual percentiles, in order to reduce misclassification [14].

We showed that quintiles of absolute income are a better predictor of SBA coverage across countries compared to relative wealth quintiles. For within country analysis, where country specific intercepts are included in the models, the two measures yield similar results in terms of goodness of fit. Even so, when both measures are included in the same model, only log-transformed absolute income remains statistically significant. We, also, showed that using the actual mean wealth index scores is worse to predict SBA coverage than the relative wealth quintiles and the absolute income. This result is particularly important for health studies comparing multiple countries, or time trend analyses, as the use of absolute income gives new perspectives on how coverage of interventions, or prevalence of an outcome changes over time, taking into account changes in group income. This approach directly answers the goal stipulated by the United Nations in 2015 [1]. Our findings confirm the usefulness of estimated income for predicting health indicators at subnational level, and as a tool for explaining whether progress may be attributed to improvements in income [3, 5, 14]. A recent article demonstrated the advantages of estimated incomes over wealth quintiles for explaining the prevalence of stunting [14].

We were able to confirm that countries with higher national incomes tend to reach universal coverage earlier, when compared to those with lower average income. In an example using countries from Sub-Saharan Africa with different levels and patterns of inequalities, we showed that Namibia - an upper-middle income country - has higher levels of SBA for all income quintiles, when compared to Ethiopia, a low-income country. These patterns (see Fig. 2) are not evident from a simple comparison of coverage by wealth quintile, without considering the estimated levels of income for each quintile.

Since the launch of the Safe Motherhood Initiative in 1987 and of the millennium development goals (MDGs) in 2000, several countries have adopted policies and strategies to improve maternal and newborn health [25, 26]. Our results can help identify which countries were most successful in increasing coverage through specific programs and initiatives, as opposed to those where increases were solely due to economic growth and rising incomes. To illustrate this point, we selected three groups of countries and compared their earliest and latest surveys according to changes in coverage and household income in the last decades.

The first group included Burkina Faso, Cambodia, Egypt, Nepal and Rwanda, the only five countries with coverage increases of over 40 percent points during a period of 10 years or more. In all those countries, the average income has increased but SBA coverage rose more rapidly than would be predicted from the change in income (Fig. 3). Such rapid coverage increases were likely due to actions other than solely the increase in income. By example, in Burkina Faso in 2006, a national subsidy policy led to a reduction in user fees for maternity services, including three goals: 1) reducing the costs of facility-based delivery care to women and their families, 2) enhancing the quality of facility-based delivery services, and 3) increasing women’s access to hospital facilities for complicated deliveries [27]. In Cambodia, programs and policies have also been put in place in the last two decades, including: 1) laws, standards and guidelines that focused specifically on ensuring political stability, supporting universal coverage with a package of high-impact interventions and mechanisms for improving coordination, 2) improved health care financing, expansion and redistribution of the health workforce (particularly midwives) and the use of data for tracking progress, and 3) increases in government allocation to health, and expansion of the health care financing schemes that included performance-based financing, health equity funds and vouchers [28]. Similarly, Egypt implemented a health sector reform program, based on five key principles: the universality, quality, equity, efficiency and sustainability. As results: 1) a new health care model was developed, based on family medicine, 2) hundreds of women’s health units, health centers and mobile clinics were set up to cover remote and deprived areas, and 3) the number of skilled health professional were increased and changes were implemented in the health system to support their work [29, 30]. In Nepal, the combination of a vast network of facilities and use of community health volunteers contributed to strengthening health systems and provide public health services free of cost; cash incentives were provided to women who delivered with SBAs, and this program also covered the cost of transportation and incentives to SBAs for attending home deliveries [31]. In Rwanda, due to decades of violence and instability, the number of SBAs was reduced markedly, but since then the government expanded human resources by using diverse programs and initiatives such as infrastructure development, decentralization of human resource management and increases in the number and quality of SBA, making these available at the village level; a comprehensive community-based health insurance scheme was also adopted to provide universal health care [32, 33]. The proactive measures taken in these five countries help explain why SBA coverage increased much faster than would be predicted by economic growth, but it should be noted that, in spite of all these efforts, in both Burkina Faso and Nepal wide inequalities persist between the richest and poorest.

Four countries showed small increases (less than 10% points) over a period of a decade or longer: Chad, Ethiopia, Nigeria and Tanzania, in spite of actions taken by their governments to improve maternal and newborn health [34–38]. In these four countries (Fig. 4), there were modest increases of income over time, but except for the wealthiest quintile in Chad and Ethiopia, SBA coverage remained virtually unchanged. Not even the wealthiest quintiles have reached universal coverage in these countries, and the wide gap between poorest and richest persist over time.

Bolivia and Haiti (Fig. 5) present a different picture, where income did not increase over more than a decade, but there were increases in SBA coverage for all quintiles, except for the wealthiest in Bolivia where universal coverage had already been reached in the earlier survey. This suggests that coverage increases are likely entirely due to policies and programs implemented at national level. For example in Bolivia, some key health policies have been adopted including increases in the health workforce, implementation of public health insurance, and investments in family medicine with a shift from traditional birth attendants to SBAs [39, 40]. Haiti, despite of decades of political, economic and natural crises, moderate increases in SBA coverage were reached in all quintiles, while income levels remained unchanged. Such progress in coverage levels may be due to programs and policies established in the country in the two decades; such as the Haitian-Cuban cooperation since 1998 [41], the Basic Emergency Obstetric Care and Comprehensive Emergency Obstetric Care (BEmOC/CEmOC) programs in 2008, among others [42].

Some limitations of our analyses need to be discussed. In terms of the attribution of dollar values to household income by quintiles, data on the relative position of household in the income distribution are not available. Thus, we could only attribute incomes based on the assumption that the relative income and asset rankings are similar. Differently from household assets that reflect accumulation of resources over longer periods of time, household income tends to be volatile over time [14]. However, we grouped household income into quintiles, which, in some extent, may reduce measurement error. Also, data on income inequality are not available for all years for each country, so we had to resort to linear interpolation to compute the Gini coefficient for missing years, what could introduce some additional measurement error. Any error should be small, though, as the Gini coefficient changes slowly over time.

The attribution of income values to asset-based quintiles also ignores possible within-country variations in purchasing power by urban/rural residence or by regions of the country. Taking these into account would involve a much more complicated strategy of ranking groups by wealth, and potentially considerably increasing classification error. We consider that the current approach, as demonstrated here, is useful while keeping the approach manageable for a large number of surveys.

Our main outcome, SBA, was chosen due to its prominence as an SDG indicator and its relevance to save deliveries. It is, however, known to be defined in a different way across countries. As stipulated by WHO in 1997, skilled attendants include doctors, nurses and midwifes [43]. In our study, we considered the definition adopted by each country, which may also include auxiliary nurses, auxiliary midwifes and community health workers, among others, according to the countries’ own programs and policies. This may lead to difficulties when comparing coverage between countries and over time, since some countries changed the cadres considered as skilled from survey to survey. Nevertheless, we inspected the time trends for each country and did not observe marked changes in coverage, even when there had been changes in the cadres regarded as being skilled. There is also the problem of self-report on who attended the delivery, as some women may be unable to provide accurate information, as described previously by Hussein et al. [44]. However, studies conducted on the validity of self-report SBA question during delivery in Kenya and Mexico concluded that this indicator is an acceptable estimate of SBA coverage [45, 46]. Given all this, we presented similar analyses for institutional delivery in the appendix, given this is an indicator considered more stable both in its definition and in reporting. It is worth noting that these two indicators presented a very high correlation based on the same datasets (*r* = 0.97; *p* < 0.001) [21].

Finally, our analyses were limited to low and middle-income countries with available data on coverage and income. We were able to include 85.7% of low income and 79.1% of middle-income countries.

Despite these limitations, our study showed that absolute income is a more precise predictor of SBA coverage across countries than is the case for wealth quintiles. These results are consistent with the previous analyses carried out by Guenther et al. [14] aimed at predicting the prevalence of stunting among under-five children.

## Conclusion

We showed that estimated absolute income levels are better predictors of SBA and institutional delivery coverage than the commonly used asset-index-based wealth quintiles, which only provide information on relative wealth within a country. Absolute income is also superior in predictive terms than the actual mean wealth index score for each quintile. We also showed that use of absolute income allows separating progress in coverage that is solely due to the population becoming wealthier, from changes that may be attributed to health policies and programs. These results are particularly important as they can help researchers and decision-makers identify countries where increased coverage is likely due to improvements other than increased income. We recommend that information on absolute income should be used more frequently for assessing national progress in health.

### Advancement/applications of the study

Use of absolute income estimates based on population surveys represents a real advancement in the understanding of social inequalities in health coverage.

## Additional files

Additional file 1: **Table S1.** National surveys for countries from 1991 to 2014. Description: *No data were available for asset indices and survey was not included in the analysis; **Country had continuous survey from 2004 to 2012. # No data were available for household income and survey was not included in the analysis. CAR: Central African Republic. (PDF 146 kb)

Additional file 2: **Table S2.** Linear regression analyses to investigate how well relative quintiles, actual mean wealth index scores and absolute income (per quintile) predict institutional delivery coverage (*N* = 1460 observations). Description: Robust standard errors in parentheses are clustered at the country level. * Income is expressed in 2011 purchasing power parity-adjusted international dollars. Model 1 and model 4: cross-country and within-country prediction of institutional delivery coverage according to wealth quintiles. Model 2 and model 5: cross-country and within-country prediction of institutional delivery coverage according to actual mean wealth scores. Model 3 and model 6: cross-country and within-country prediction of institutional delivery coverage according to household income. Model 7: within-country prediction of institutional delivery coverage according to wealth quintiles and household income. **Figure S1.** Institutional delivery coverage by log absolute income. Each dot is one quintile in each survey. **Figure S2.** Institutional delivery coverage in Namibia, Nigeria and Ethiopia according to a) wealth quintiles and b) absolute income in the most recent survey. **Figure S3.** Five countries with increases in institutional delivery coverage > = 40 percentage points over time: Cambodia, Egypt, Indonesia, Nepal and Rwanda. **Figure S4.** Four countries with increases in institutional delivery coverage < 10 percentage points over 10 or more years: Central African Republic (CAR), Ethiopia, Madagascar and Tanzania. **Figure S5.** Two countries with no progress in household income over 10 or more years but with increase in institutional delivery coverage (Bolivia, Haiti). (PDF 812 kb)

Additional file 3: Additional file Data availability. (XLSX 82 kb)

## Abbreviations

BEmOC: Basic emergency obstetric care
CEmOC: Comprehensive emergency obstetric care
DHS: Demographic and Health Surveys
LMICs: Low and middle-income countries
MDG: Millennium development goal
MICS: Multiple Indicator Cluster Surveys
RHS: Reproductive Health Surveys (RHS)
SBA: Skilled birth attendant/attendance
SD: Standard deviation
SDGs: Sustainable development goals
SEP: Socioeconomic position
