# Supplementary material for: Absolute income is a better predictor of coverage by skilled birth attendance than relative wealth quintiles in a multicountry analysis: comparison of 100 low- and middle-income countries
Source: BMC Pregnancy Childbirth. 2018 Apr 16;18:104. doi: 10.1186/s12884-018-1734-0 (PMC5902965; doi:10.1186/s12884-018-1734-0)
Supplement: Supplementary file 1 — Table S1. National surveys for countries from 1991 to 2014. Description: *No data were available for asset indices and survey was not included in the analysis; **Country had continuous survey from 2004 to 2012. # No data were available for household income and survey was not included in the analysis. CAR: Central African Republic. (PDF 146 kb) [file 12884_2018_1734_MOESM1_ESM.pdf]

Country	Earliest survey	2nd survey	3rd survey	4th survey	5th survey	6th survey	7th/8th surveys	Country	Earliest survey	2nd survey	3rd survey	4th survey	5th survey	6th survey
Afghanistan	2010							Lesotho	2004	2009	2014			
Albania	2002	2005	2008					Liberia	2007	2013				
Algeria	2012							Macedonia	2005	2011				
Armenia	2000	2005	2010					Madagascar	1997	2003	2008			
Azerbaijan	2006							Malawi	2000	2004	2006	2010	2013	
Bangladesh	1993	1996	1999	2004	2007	2011	2012 & 2014	Maldives	2009					
Barbados	2012							Mali	1995	2001	2006	2012		
Belarus	2005	2012						Mauritania	2007	2011				
Belize	1991	2006	2011					Moldova	2005	2012				
Benin	1996	2001	2006	2011				Mongolia	2005	2010	2013			
Buthan	2010							Montenegro	2005	2013				
Bolivia	1994	1998	2003	2008				Morocco	2003					
Bosnia &Herz.	2006	2011						Mozambique	1997	2003	2008	2011		
Brazil	1996	2006						Namibia	2000	2006	2013			
Burkina_Faso	1998	2003	2006	2010				Nepal	1996	2001	2006	2011	2014	
Burundi	2005	2010						Nicaragua	1998	2001	2006			
Cambodia	2000	2005	2010	2014				Niger	1998	2006	2012			
Cameroon	1998	2004	2006	2011				Nigeria	1999*	2003	2007	2008	2011	2013
CAR	1994	2006	2010					Pakistan	2006	2012				
Chad	1996	2004	2010	2014				Panama	2013					
Colombia	1995	2000	2005	2010				Paraguay	1995	1998	2004	2008		

Comoros	1996	2012						Philippines	1993	1998	2003	2008	2013	
Congo_Brazzaville	2005	2011						Peru	1996	2000	2004/2012**			
Congo_D.R.	2007	2010	2013					Rwanda	2000	2005	2010	2014		
Costa_Rica	1992	2011						São_Tome_&_P.	2008	2014				
Cote_dIvoire	1994	1998	2006	2011				Senegal	1997	2005	2010	2012	2014	
Dominican_Rep.	1996	1999	2002	2007	2013	2014		Serbia	2005	2010	2014			
Ecuador	1994	1999	2004					Sierra_Leone	2005	2008	2010	2013		
Egypt	1995	2000	2005	2008	2014			South_Sudan	2010					
El_Savador	2014							St_Lucia	2012					
Ethiopia	2000	2005	2011					Sudan	2010	2014				
Gabon	2000	2012						Suriname	2006	2010				
Gambia	2005	2013						Swaziland	2006	2010	2014			
Georgia	2005							Tajikistan	2005	2012				
Ghana	1993	1998	2003	2006	2008	2011	2014	Tanzania	1996	1999	2004	2010		
Guatemala	1995	1998	2008					Thailand	2005	2012				
Guinea	1999	2005	2012					Timor_Leste	2009					
Guinea_Bissau	2006	2014						Togo	1998	2006	2010	2013		
Guyana	2006	2009	2014					Trinidad_and_Tobago	2006					
Haiti	1994#	2000	2005	2012				Tunisia	2011					
Honduras	2005	2011						Turkey	1993	1998	2003			
India	1998	2005						Turkemistan	2006					
Indonesia	1997	2002	2007	2012				Uganda	1995	2000	2006	2011		
Iraq	2006*	2011						Ukraine	2005	2012				

Jamaica	2005*	2011						Uruguay	2012				
Jordan	1997	2002	2007	2012				Uzbekistan	1996	2006			
Kazakhstan	1995	1999	2006	2010				Vietnam	1997	2002	2006	2010	2013	
Kenya	1993	1998	2003	2008	2014			Yemen	2006	2013				
Kyrgyzstan	1997	2006	2012	2014				Zambia	1996	2001	2007	2013		
Lao	2006	2011						Zimbabwe	1994	1999	2005	2009	2010	2014
