# Supplementary material for: Absolute income is a better predictor of coverage by skilled birth attendance than relative wealth quintiles in a multicountry analysis: comparison of 100 low- and middle-income countries
Source: BMC Pregnancy Childbirth. 2018 Apr 16;18:104. doi: 10.1186/s12884-018-1734-0 (PMC5902965; doi:10.1186/s12884-018-1734-0)
Supplement: Supplementary file 2 — Table S2. Linear regression analyses to investigate how well relative quintiles, actual mean wealth index scores and absolute income (per quintile) predict institutional delivery coverage (N = 1460 observations). Description: Robust standard errors in parentheses are clustered at the country level. * Income is expressed in 2011 purchasing power parity-adjusted international dollars. Model 1 and model 4: cross-country and within-country prediction of institutional delivery coverage according to wealth quintiles. Model 2 and model 5: cross-country and within-country prediction of institutional delivery coverage according to actual mean wealth scores. Model 3 and model 6: cross-country and within-country prediction of institutional delivery coverage according to household income. Model 7: within-country prediction of institutional delivery coverage according to wealth quintiles and household income. Figure S1. Institutional delivery coverage by log absolute income. Each dot is one quintile in each survey. Figure S2. Institutional delivery coverage in Namibia, Nigeria and Ethiopia according to a) wealth quintiles and b) absolute income in the most recent survey. Figure S3. Five countries with increases in institutional delivery coverage > = 40 percentage points over time: Cambodia, Egypt, Indonesia, Nepal and Rwanda. Figure S4. Four countries with increases in institutional delivery coverage < 10 percentage points over 10 or more years: Central African Republic (CAR), Ethiopia, Madagascar and Tanzania. Figure S5. Two countries with no progress in household income over 10 or more years but with increase in institutional delivery coverage (Bolivia, Haiti). (PDF 812 kb) [file 12884_2018_1734_MOESM2_ESM.pdf]

Web appendix Table and Figures

	Institutional delivery coverage (coefficients expressed as percent point)						
**Analysis level**	**Cross-country analysis**			**Within country analysis**			
	Model 1	Model2	Model 3	Model 4	Model 5	Model 6	Model 7
Asset quintile 1	0 (reference) p <0.001			0 (reference) p <0.001			0 (reference) p=0.320
Asset quintile 2	10.03 (0.99)			10.03 (1.11)			0.16 (3.85)
Asset quintile 3	18.52 (1.80)			18.52 (2.01)			2.14 (6.37)
Asset quintile 4	28.34 (2.22)			28.34 (2.48)			5.40 (9.03)
Asset quintile 5	40.54 (2.53)			40.54 (2.82)			5.74 (13.80)
Mean wealth scores		7.14 (1.83) P<0.001			6.86 (1.94) P<0.001		
Log income*			19.46 (1.37) p<0,001			18.68 (1.26) p<0.001	15.74 (6.08) p=0.011
Survey specific intercepts	NO	NO	NO	YES	YES	YES	YES
R-squared	0.200	0.120	0.475	0.896	0.804	0,901	0,902

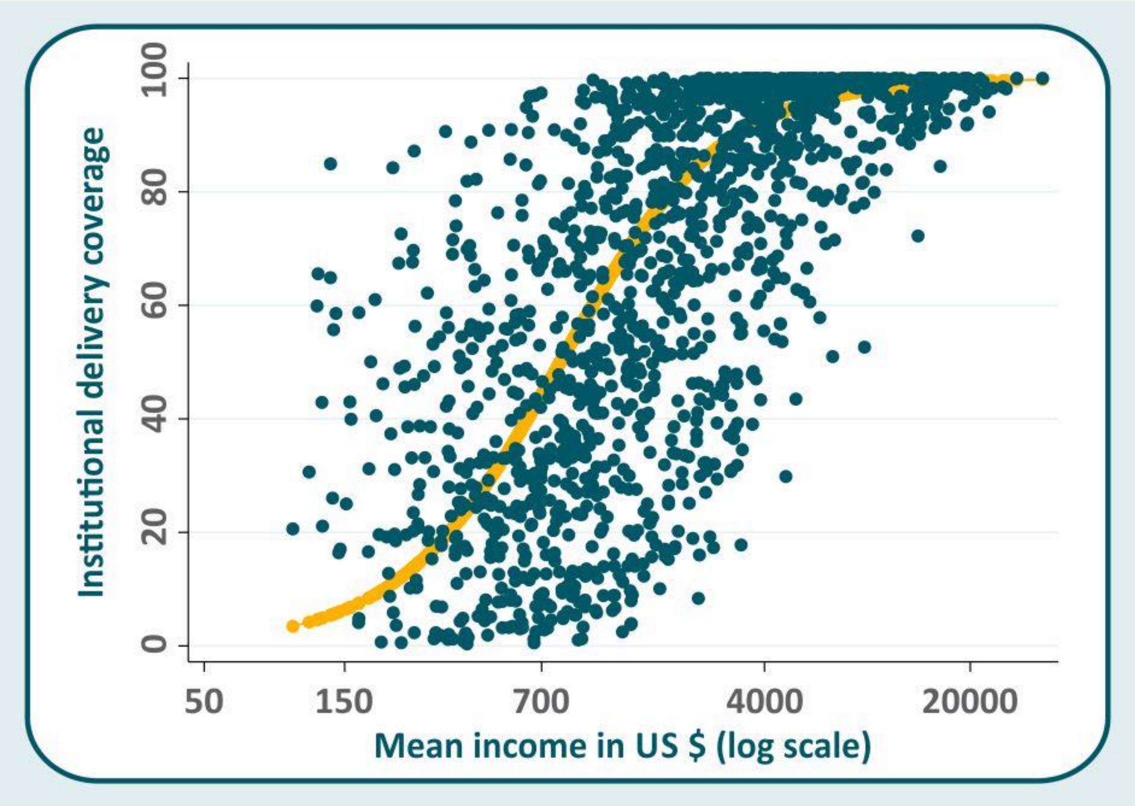
Institutional delivery coverage
0
20
40
60
80
100
50
150
700
4000
20000
Mean income in US $ (log scale)

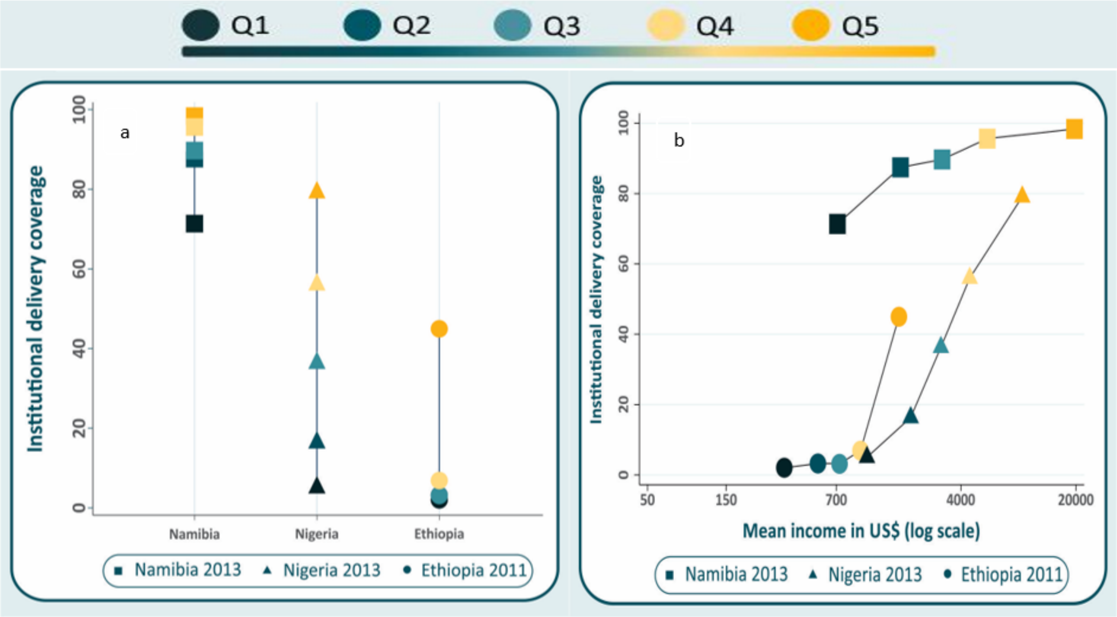
Q1
Q2
Q3
Q4
Q5
a
Institutional delivery coverage
100
80
60
40
20
0
Namibia
Nigeria
Ethiopia
Namibia 2013
Nigeria 2013
Ethiopia 2011
b
Institutional delivery coverage
100
80
60
40
20
0
50
150
700
4000
20000
Mean income in US$ (log scale)
Namibia 2013
Nigeria 2013
Ethiopia 2011

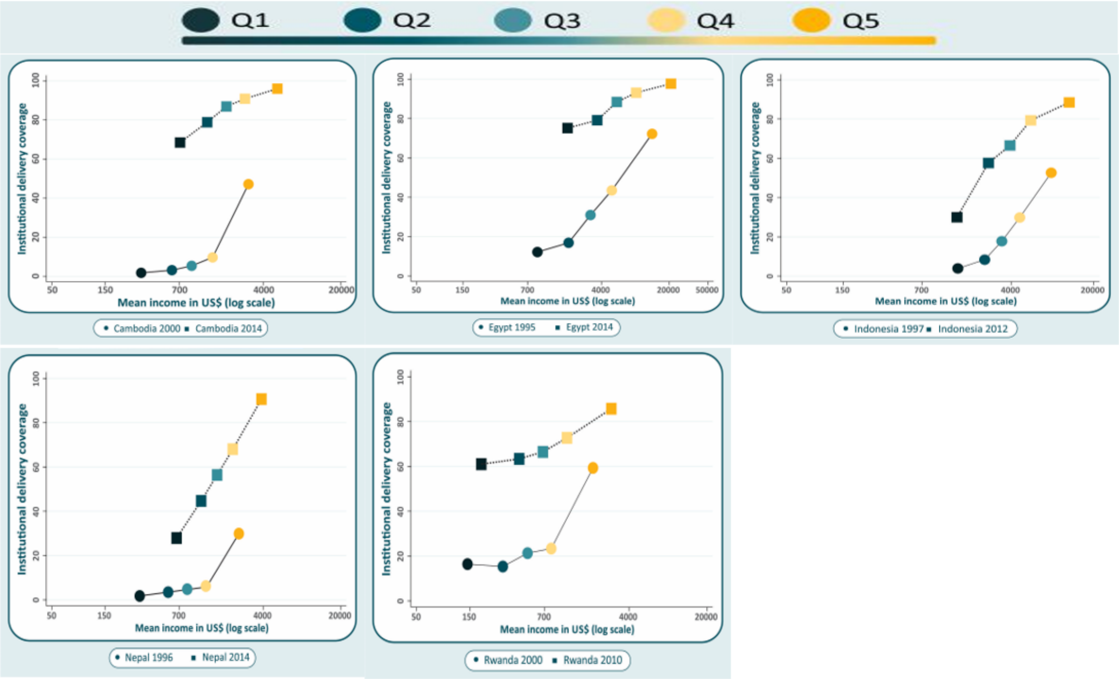

Q1
Q2
Q3
Q4
Q5
Institutional delivery coverage
Mean income in US$ (log scale)
Cambodia 2000
Cambodia 2014
Egypt 1995
Egypt 2014
Indonesia 1997
Indonesia 2012
Nepal 1996
Nepal 2014
Rwanda 2000
Rwanda 2010

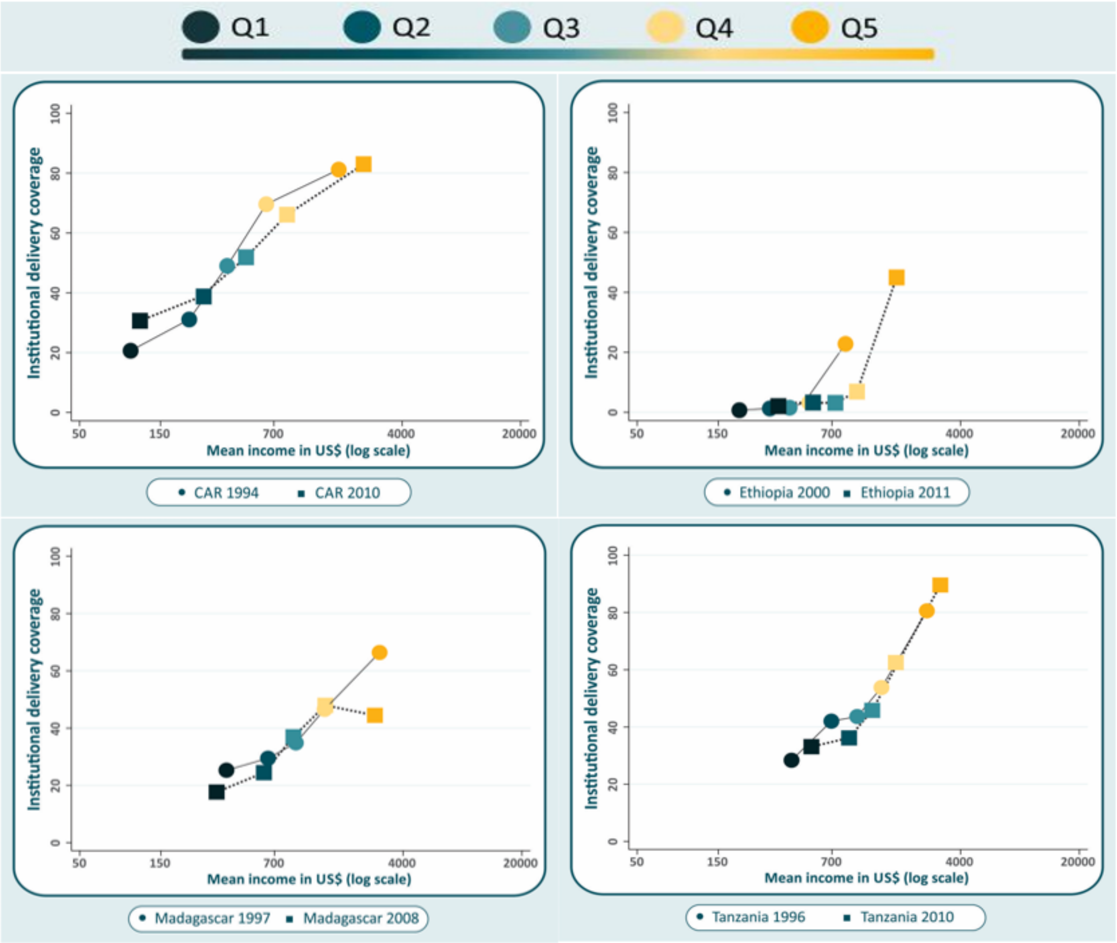

Q1
Q2
Q3
Q4
Q5
Institutional delivery coverage
Mean income in US$ (log scale)
CAR 1994
CAR 2010
Ethiopia 2000
Ethiopia 2011
Madagascar 1997
Madagascar 2008
Tanzania 1996
Tanzania 2010

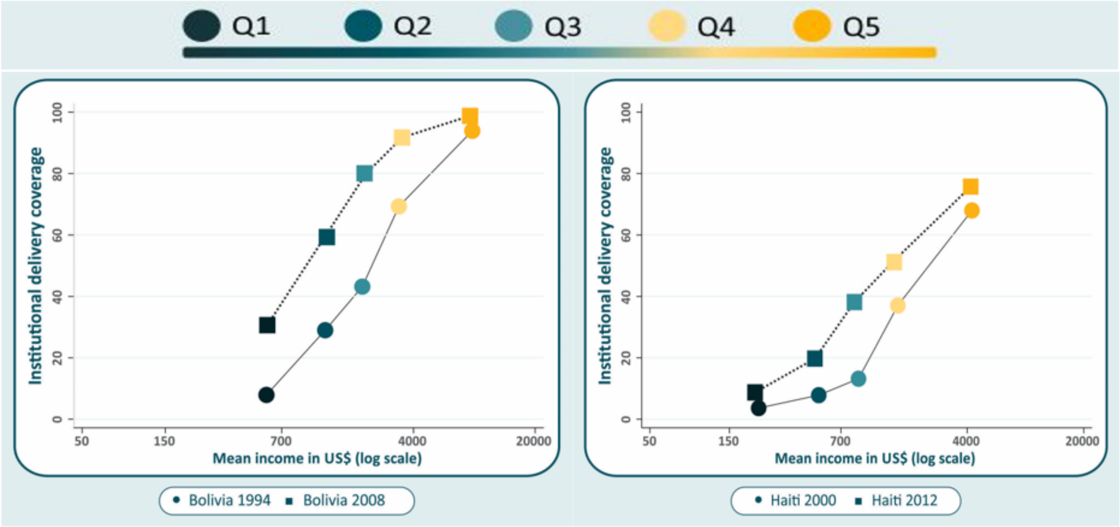
Q1
Q2
Q3
Q4
Q5
Institutional delivery coverage
0
20
40
60
80
100
50
150
700
4000
20000
Mean income in US$ (log scale)
Bolivia 1994
Bolivia 2008
Institutional delivery coverage
0
20
40
60
80
100
50
150
700
4000
20000
Mean income in US$ (log scale)
Haiti 2000
Haiti 2012
